# Quantum Sensing of Local Magnetic Phase Transitions and Fluctuations near the Curie Temperature in Tm_3_Fe_5_O_12_ Using NV Centers

**DOI:** 10.3390/mi16060643

**Published:** 2025-05-28

**Authors:** Yuqing Zhu, Mengyuan Cai, Qian Zhang, Peiyang Wang, Yuanjie Yang, Jiaxin Zhao, Wei Zhu, Guanzhong Wang

**Affiliations:** 1 Department of Physics, University of Science and Technology of China, Hefei 230026, China; zhuyuqing@mail.ustc.edu.cn (Y.Z.); mycai@mail.ustc.edu.cn (M.C.); qianzhang@mail.ustc.edu.cn (Q.Z.); yangyj@mail.ustc.edu.cn (Y.Y.); jxzhao@mail.ustc.edu.cn (J.Z.); zhuw@ustc.edu.cn (W.Z.); 2 Key Laboratory of Strongly-Coupled Matter Physics, Chinese Academy of Sciences, Hefei 230026, China

**Keywords:** nitrogen-vacancy center, quantum magnetometry, magnetic phase transition, thulium iron garnet, Hall effect

## Abstract

Thulium iron garnet (Tm_3_Fe_5_O_12_, TmIG) is a promising material for next-generation spintronic and quantum technologies owing to its high Curie temperature and strong perpendicular magnetic anisotropy. However, conventional magnetometry techniques are limited by insufficient spatial resolution and sensitivity to probe local magnetic phase transitions and critical spin dynamics in thin films. In this study, we present the first quantitative investigation of local magnetic field fluctuations near the Curie temperature in TmIG thin films using nitrogen-vacancy (NV) center-based quantum sensing. By integrating optically detected magnetic resonance (ODMR) and NV spin relaxometry (T_1_ measurements) with macroscopic techniques such as SQUID magnetometry and Hall effect measurements, we systematically characterize both the static magnetization and dynamic spin fluctuations across the magnetic phase transition. Our results reveal a pronounced enhancement in NV spin relaxation rates near 360 K, providing direct evidence of critical spin fluctuations at the nanoscale. This work highlights the unique advantages of NV quantum sensors for investigating dynamic critical phenomena in complex magnetic systems and establishes a versatile, multimodal framework for studying local phase transition kinetics in high-temperature magnetic insulators.

## 1. Introduction

Recent advances in quantum materials have driven significant progress in spintronics and quantum technologies, with ferrite magnets like Tm_3_Fe_5_O_12_ (TmIG) attracting attention due to their high Curie temperatures and strong magnetic anisotropy [1,2,3,4]. These properties make TmIG an excellent platform for investigating high-temperature spintronic devices and magnetic memory systems [5,6,7,8]. However, characterizing such materials at the nanoscale remains challenging, as conventional techniques—including SQUID magnetometry and MFM—suffer from limited spatial resolution, thermal noise interference, and stringent operational conditions [9,10,11,12].

Solid-state quantum sensors, particularly nitrogen-vacancy (NV) centers in diamond, have emerged as transformative tools for nanoscale magnetism research [13,14,15]. Their unique advantages, such as ambient-temperature operation, noninvasive detection, and compatibility with extreme environments, enable precise mapping of both static and dynamic magnetic fields in complex systems [16,17,18]. Complementarily, Hall effect measurements provide critical insights into spin transport and magnetic phase transitions through electrical readouts [19].

Here, we integrate NV quantum magnetometry with Hall transport characterization to systematically investigate TmIG’s magnetic behavior across its Curie temperature. Importantly, this study represents the first quantitative investigation of dynamic magnetic field fluctuations in the critical region of the phase transition in TmIG thin films, providing new insights into local spin dynamics near *T_C_*. NV ensemble measurements reveal temperature-dependent magnetization dynamics, while Hall resistance analysis uncovers magnetic anomalies near the phase transition. This dual-modality approach not only resolves the limitations of traditional characterization methods but also establishes a universal framework for probing high Curie temperature materials, advancing both fundamental understanding and device engineering in quantum magnetism.

## 2. Materials and Methods

### 2.1. Sample and Diamond NV Sensor Preparation

Tm_3_Fe_5_O_12_ (TmIG) thin films were fabricated using an AJA RF magnetron sputtering system with a 3-inch diameter TmIG ceramic target (99.9% purity) onto 10 × 10 mm^2^ Gd_3_Ga_5_O_12_ (111) single-crystal substrates (lattice constant: 12.383 Å). Prior to deposition, the substrates were ultrasonically cleaned for 10 min sequentially in acetone, isopropanol, and deionized water, then dried with high-purity nitrogen and immediately loaded into the sputtering chamber, which was evacuated to a base pressure below 5 × 10⁻⁷ Torr. During deposition, the substrate temperature was maintained at 700 °C with an RF power of 100 W, a working pressure of 1.5 Pa, and an Ar/O_2_ flow ratio of 4:1. The target-to-substrate distance was set at 10 cm. The target was pre-sputtered for 20 min to remove surface contaminants and stabilize the sputtering conditions. The film growth rate was approximately 2.5 nm/min, resulting in a film thickness of approximately 20 nm. After deposition, the films were in situ annealed at 700 °C for 10 min and subsequently cooled to room temperature at a rate of 50 °C/min to enhance crystallinity and reduce oxygen vacancy concentration.

The diamond NV sensor was prepared by first growing high-purity single-crystal [100]-oriented diamond via chemical vapor deposition (CVD), followed by cutting and mechanical polishing to obtain a [111]-oriented substrate, thereby optimizing magnetic field sensitivity for subsequent measurements. Shallow NV centers were created by implanting ^14^N⁺ ions at an energy of 5 keV and a dose of 10^13^ ions/cm^2^, resulting in an NV layer located approximately 7 nm beneath the diamond surface. Post-implantation annealing was conducted at 800 °C for 2 h under a vacuum of 5 × 10⁻⁵ Pa to promote vacancy–nitrogen complex formation. Finally, the diamond was cleaned using a mixed acid solution of concentrated H_2_SO_4_, HNO_3_, and HClO_4_ (1:1:1 by volume) at 220 °C for 2 h to remove surface graphitic carbon and residual contaminants. The resulting [111]-oriented, shallow NV-doped diamond exhibited a uniform NV distribution, high optical quality, and excellent quantum magnetic sensing performance.

### 2.2. Magnetic Property and Electrical Transport Measurements

The magnetic and transport properties of the Tm_3_Fe_5_O_12_ films were systematically investigated under controlled and reproducible conditions.

#### 2.2.1. Magnetometry Measurements

Magnetization hysteresis (M-H) loops were measured at 300 K using a Quantum Design MPMS3 system in vibrating sample magnetometer (VSM) mode. For M-H characterization, samples were mounted using plastic drinking straws and placed perpendicular to the applied magnetic field (out-of-plane geometry). The magnetic field was swept from −20,000 Oe to +20,000 Oe, with a 10 Oe step size near zero field for detailed coercivity resolution, and 100 Oe steps at higher fields. The magnetization signal at each field was recorded after stabilization (dwell time ≥ 2 s).

Temperature-dependent magnetization (M-T) curves were acquired on the same system in field-cooled (FC) mode, with the sample mounted on a clean quartz rod to minimize background. Measurements were performed under a constant out-of-plane field of 5 kOe from 300 K to 380 K in 0.5 K increments. At each temperature, the sample was stabilized for at least 2 min before measurement, and each data point was averaged over three independent runs to ensure reproducibility.

#### 2.2.2. Electrical Transport Measurements

Hall bar devices (channeldimensions: 200 µm × 20 µm) were fabricated via photolithography and Ar ion milling. Longitudinal and transverse resistivities were measured in a Quantum Design PPMS system (five-probe configuration), with excitation currents of 100 µA unless otherwise specified. Resistivity and Hall data were collected from 2 K to 360 K at 1 K intervals; each point represents an average of three measurements. Hall voltages were recorded under alternating magnetic field polarities to suppress thermoelectric contributions. Prior to each measurement, thermal equilibration was ensured for at least 1 min. The resulting data were further processed using the Savitzky–Golay smoothing algorithm to enhance signal quality and resolution.

### 2.3. Diamond NV Magnetometry Experimental Platform

**Figure 1:**
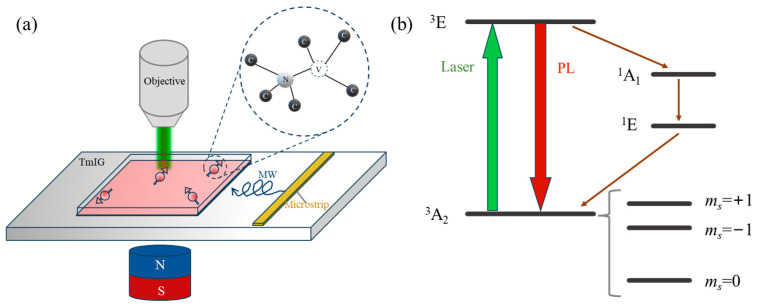
Schematic of the experimental setup and diamond NV level structure. (**a**) A diamond with NV centers is placed on a thulium iron garnet (Tm_3_Fe_5_O_12_, thickness: 20 nm) thin film. A 532 nm green laser with a power of 0.8 mW is used, and red fluorescence is collected through the same objective. Microwave (MW) radiation is generated through a microwave line, and a neodymium iron boron permanent magnet provides the external magnetic field. Inset: atomic structure of the NV centers in the diamond; (**b**) initialization and readout of the NV spin are achieved through green laser excitation and red photoluminescence (PL) detection. There are two pathways for returning from the excited state to the ground state: one is direct radiative fluorescence to the ground state, and the other is returning to the ground state through an intermediate state without radiating fluorescence. The electronic spin ground state of the NV center is a triplet with spin quantum numbers *m_s_* = 0 and *m_s_* = ±1.

A custom diamond NV magnetometry platform was developed for high-sensitivity, noninvasive measurement of local magnetic fields in Tm_3_Fe_5_O_12_ thin films, as schematically shown in Figure 1a. The [111]-oriented diamond containing a shallow NV ensemble was placed in direct contact with the sample. Optical excitation (532 nm) and fluorescence detection were achieved via a high-numerical-aperture objective (NA = 0.9), which enables efficient collection of NV center fluorescence and provides submicron spatial resolution. Microwave (MW) fields were delivered via a 30 μm copper wire, and a tunable external magnetic bias was generated by a movable NdFeB permanent magnet. Laser excitation was delivered from the diamond backside to accommodate substrate thickness and maximize fluorescence collection efficiency.

In our confocal NV-based magnetometry setup, the lateral spatial resolution is determined by the optical diffraction limit of the objective (NA = 0.9, λ = 532 nm), yielding a spot size of approximately 360 nm. This means that each measurement probes the local magnetic field within a submicron-scale volume. The depth sensitivity is governed by the average NV implantation depth, which in this study is approximately 7 nm beneath the diamond surface. While the current experimental platform operates in a point-by-point mode without full 2D mapping or variable-depth profiling, this approach still provides substantial advantages for local magnetic characterization compared to conventional techniques.

The diamond NV center’s ground state spin triplet (***S*** = 1) enables vector magnetic sensing by optically detected magnetic resonance (ODMR). The presence of an external magnetic field leads to Zeeman splitting of the *m_s_* = ±1 states, described by the following Hamiltonian (the energy levels and working principle are illustrated in Figure 1b) [20]:

(1) $$H=DS_{z}^{2}+E\left(S_{x}^{2}-S_{y}^{2}\right)+\gamma_{e}B\cdot S$$

where *D* is the zero-field splitting, *E* denotes strain-induced splitting, *γ_e_* = 2.8 MHz/G is the electron gyromagnetic ratio, ***B*** is the external magnetic field, and ***S*** is the electron spin operator. The spin states of the NV center can be optically initialized and read out, with spin-selective nonradiative decay processes leading to spin-state-dependent photoluminescence intensity, enabling spin-state discrimination (as shown in Figure 1b).

By applying a microwave field with a frequency resonant with the energy splitting between the *m_s_* = 0 and *m_s_* = ±1 spin states, ODMR enables determination of the static magnetic field’s magnitude and orientation. When the external magnetic field is oriented along the [111] direction, the Zeeman splitting between the two outer ODMR resonances is as follows [21]:

(2) $$\Delta v=2\gamma_{e}B_{NV}$$

where Δ*ν* is the frequency separation between the ODMR dips and *B_NV_* is the magnetic field projection along the NV axis. Thus, the local field can be obtained straightforwardly as follows:

(3) $$B_{NV}=\frac{\Delta v}{2\gamma_{e}}$$

System sensitivity (~1.6 μT/√Hz) is established by fitting the ODMR spectrum under typical conditions. Accurate temperature calibration is achieved by tracking the zero-field splitting parameter *D* as a function of temperature. In our experiments, multiple reference point measurements showed that variations in *D* remained within 0.1 MHz, corresponding to a temperature drift rate below 1.5 K/h. This demonstrates excellent thermal stability of the system, with reference point control ensuring thermal uniformity throughout all measurements. All experimental measurements—both static and dynamic—are synchronized through integrated hardware and software control.

This highly integrated system enables quantitative measurement of static and dynamic magnetic properties of Tm_3_Fe_5_O_12_ thin films over a wide temperature range, enabling detailed investigations of magnetic phase transitions and local magnetic phenomena at the microscale.

## 3. Results

### 3.1. Structural and Compositional Characterization of TmIG Films

**Figure 2:**
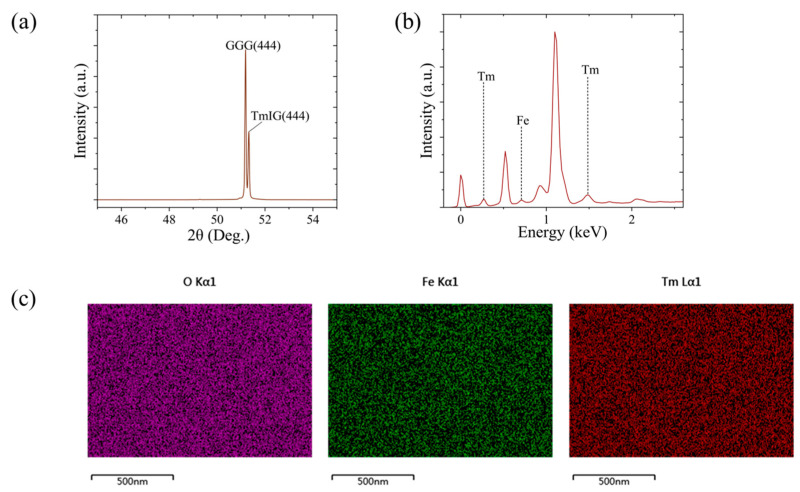
Structural and compositional characterization of Tm_3_Fe_5_O_12_/Gd_3_Ga_5_O_12_(111) thin films. (**a**) XRD 2θ–ω scan; (**b**) EDS elemental composition analysis; and (**c**) SEM-EDS elemental mapping.

Tm_3_Fe_5_O_12_ is a garnet-type oxide with a cubic lattice structure in which the magnetism arises primarily from the ordered arrangement of Fe^3^⁺ ions and their interaction with rare-earth Tm^3^⁺ ions [22,23]. Figure 2 presents the structural and compositional characterization of TmIG films grown on Gd_3_Ga_5_O_12_ (111) substrates. The X-ray diffraction (XRD) 2θ–ω scan shown in Figure 2a reveals distinct, sharp peaks exclusively from TmIG and the GGG substrate, with no impurity phases detected, confirming high phase purity and crystalline quality. Notably, the TmIG (444) peak centers at 51.32°, exhibiting an FWHM less than 0.05°, indicating the high crystalline quality of the film. Lattice parameters derived from peak positions are 12.33 Å for the film and 12.35 Å for the substrate, yielding a minor in-plane lattice mismatch of −0.16%. Figure 2b displays the energy-dispersive X-ray spectroscopy (EDS) results, revealing that the measured elemental ratios are consistent with the stoichiometric composition, with deviations less than 2%. The scanning electron microscopy–energy dispersive X-ray spectroscopy (SEM-EDS) elemental mapping in Figure 2c demonstrates a uniform spatial distribution of Tm, Fe, and O throughout the film and substrate, with no observable aggregation or deficiency. These findings establish the high structural integrity and chemical uniformity of our TmIG films, ensuring a solid base for ensuing magnetic and NV sensing investigations.

### 3.2. Basic Properties of Magnetism and Electricity in TmIG Films

**Figure 3:**
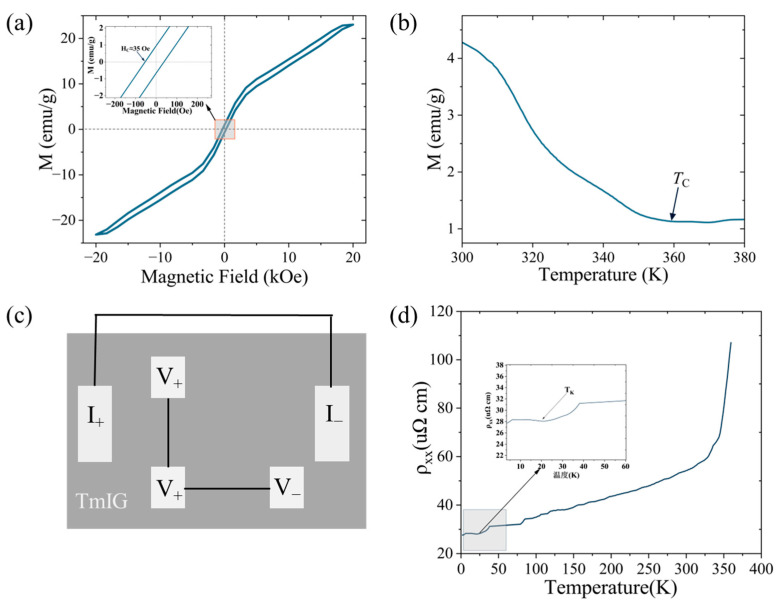
Magnetic and electrical transport properties of Tm_3_Fe_5_O_12_. (**a**) M-H curve at 300 K. The inset shows an enlarged view around the coercive field. The arrow in the inset indicates the intercept on the horizontal axis, corresponding to the coercivity (*H*c) of 35 Oe; (**b**) the M-T curve at 5000 Oe. The arrow indicates the position of the estimated Curie temperature (*T_C_*). Note: The difference in absolute magnetization values between M-H and M-T measurements is due to variations in sample mounting and experimental procedure, as detailed in Section 2.2; (**c**) schematic diagram of the Tm_3_Fe_5_O_12_ Hall bar device; and (**d**) longitudinal resistivity versus temperature for the Tm_3_Fe_5_O_12_ Hall bar device, where T_K_ represents the Kondo temperature.

After confirming the high structural quality and compositional uniformity of the TmIG thin films, we systematically investigated their key magnetic and electrical transport properties. As shown in Figure 3a, the M-H loop measured at 300 K displays a hysteresis loop, with a coercivity of 35 Oe. These features demonstrate robust ferromagnetism and strong perpendicular magnetic anisotropy, which are both attributable to the optimized fabrication process and a uniform magnetic domain structure with minimal pinning.

Figure 3b presents the M-T curve, which demonstrates a smooth second-order ferromagnetic–paramagnetic transition near 360 K, in good agreement with theoretical expectations and with the requirements for spintronic and quantum sensing applications.

The electrical transport properties are shown in Figure 3c,d, where the device exhibits quasi-metallic resistivity behavior between 50 and 300 K, clear Kondo scattering at low temperatures (~20 K), and a prominent resistivity upturn near the Curie temperature (*T_C_*) due to enhanced magnetic fluctuations [24]. These comprehensive magnetic and transport assessments provide a strong foundation for the subsequent NV-center-based local phase transition studies.

Overall, TmIG thin films exhibit excellent ferromagnetic and tunable electrical properties, together with evident magnetic fluctuation features near the Curie point, thus serving as an ideal platform for subsequent NV-center-based local magnetic phase transition investigations.

### 3.3. Local Magnetic Detection Result by Diamond NV Centers

#### 3.3.1. Local Static Magnetization Measurement via Diamond NV Centers

**Figure 4:**
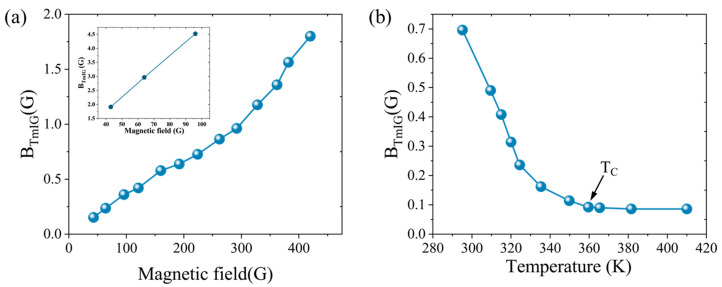
Field and temperature dependence of Tm_3_Fe_5_O_12_ magnetization detected by NV centers. (**a**) Variation in B_TmIG_ with external magnetic field strength (inset shows a magnified view near zero fields); (**b**) dependence of B_TmIG_ on temperature. The arrow indicates the position of the Curie temperature (*T_C_*).

In this section, high-resolution local out-of-plane static magnetic field measurements of Tm_3_Fe_5_O_12_ thin films were performed using diamond NV center quantum probes. First, at room temperature, the local magnetic field distribution was recorded under various external magnetic fields, as shown in Figure 4a. As B_tot_ increased, the out-of-plane magnetic field B_TmIG_ monotonically increased, and exhibited a clear linear response in the weak field regime (B_tot_ < 100 G), with the inset near zero field further emphasizing this linearity. These results confirm the capability of NV-based sensing for precise local magnetic field measurements in TmIG films.

Subsequently, under a fixed external field of B_tot_ ≈ 250 G, the temperature dependence of the local magnetic field was measured, as shown in Figure 4b. With the temperature increasing from room temperature to around 360 K, the out-of-plane field dropped sharply from 0.7 G to about 0.05 G, indicating the ferromagnetic–paramagnetic transition. Above the Curie temperature, the residual local field remained at roughly 0.05 G. The measured data clearly captured the temperature evolution of local magnetization, laying a foundation for subsequent analysis of phase transition dynamics and fluctuation mechanisms.

#### 3.3.2. Detection of Critical Magnetic Fluctuations via NV Spin Relaxation

To investigate dynamic magnetic fluctuations, we employed NV-center-based *T*_1_ relaxometry based on *T*_1_ relaxation, which is exquisitely sensitive to low-frequency magnetic noise generated by fluctuating spins near the phase transition.

**Figure 5:**
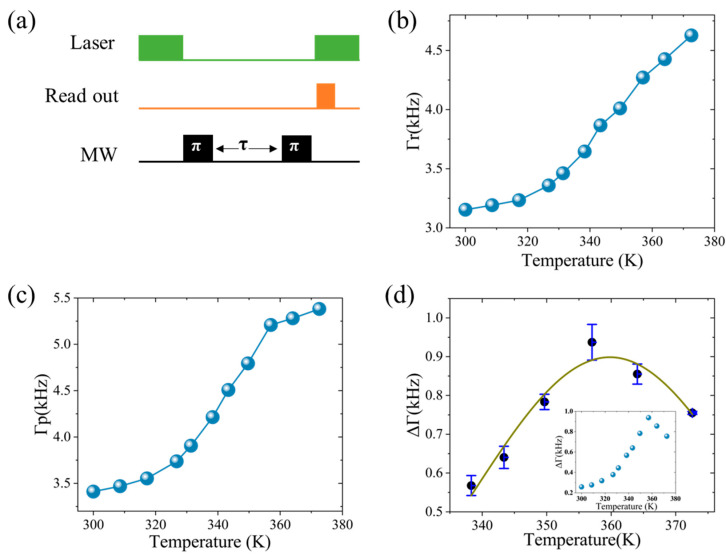
Detection of magnetic spin fluctuations in Tm_3_Fe_5_O_12_ using spin relaxation. (**a**) Schematic of T_1_ spin relaxation pulse sequence used for probing magnetic fluctuations (MW: microwave); (**b**) temperature-dependent relaxation rate measured at a reference location away from the sample; (**c**) relaxation rate at the NV probe site adjacent to the TmIG film; and (**d**) net relaxation rate with Gaussian fit in the critical region (inset: variation in the net relaxation rate over the full temperature range).

Figure 5a illustrates the employed *T*_1_ pulse sequence. We measured relaxation at both a probe location adjacent to the TmIG surface and at a remote reference under a constant external magnetic field. As shown in Figure 5b,c, the relaxation rate at the reference site (ΔΓr) increases moderately with temperature, consistent with phonon-mediated relaxation. At the probe site (ΔΓp), however, a pronounced peak appears near the Curie temperature (~360 K), significantly surpassing the reference values.

By subtracting the relaxation rate measured at the remote reference site (Γᵣ) from that near the TmIG sample surface (Γp), we obtained the net relaxation rate ΔΓ = Γr − Γp, isolating local magnetic fluctuations originating specifically from the TmIG film. Within the 300–340 K temperature range, ΔΓ varies modestly by about 0.3 kHz (see inset of Figure 5d for the full temperature span), indicating that magnetic fluctuations remain in a quasi-static equilibrium state. We focus on the critical region near the Curie temperature (340–375 K), where the finely resolved ΔΓ data in Figure 5d exhibit a pronounced peak.

This peak, occurring between 355 K and 365 K with a maximum near 360 K, closely aligns with the Curie temperature independently determined by MPMS measurements. The sharp increase in ΔΓ in this range signals enhanced local magnetic fluctuations, resulting in elevated low-frequency magnetic noise detected by the proximal NV centers and thereby causing a significant enhancement of their *T*_1_ spin relaxation.

To quantitatively analyze this behavior, the temperature-dependent net relaxation rate ΔΓ(*T*) within the critical region was fitted using a Gaussian fluctuation model described as follows:

(4) $$\Delta \Gamma \left(T\right)=\Gamma_{0}\exp\left(-\frac{T-T_{c}}{2\sigma^{2}}\right)$$

The fit yields a peak fluctuation rate Γ_0_ = 0.899 ± 0.026 kHz, a width parameter σ = 21.4 ± 1.9 K, and a critical temperature *T_C_* = 359.8 ± 1.1 K, with a goodness-of-fit R^2^ > 0.99. These results quantitatively confirm that critical magnetic fluctuations near the Curie temperature (*T_C_*) dominate the NV spin relaxation process in this temperature range. The uncertainties represent standard errors derived from the covariance matrix of the fit.

This result offers a quantitative probe of the dynamical critical behavior at the nanoscale: below *T_C_*, the fluctuations are suppressed due to long-range order; as *T_C_* is approached, slow collective spin fluctuations are strongly coupled to the NV centers, enhancing relaxation; and above *T_C_*, fluctuations decline with loss of magnetic order. Our findings provide compelling experimental evidence for local critical dynamics, with significant implications for fundamental studies and future device applications based on quantum magnetic sensing.

#### 3.3.3. Measurement Reliability and Error Analysis

The primary source of uncertainty in static field measurements arises from the NV sensor’s magnetic field resolution (approximately ±0.3 μT), improved by repeated averaging. Temperature stability is ensured by tracking the zero-field splitting, with a measured thermal drift of less than 1.5 K/h and spatial localization errors below 0.5 μm. For NV spin relaxometry, repeated signal accumulation and background subtraction yield standard errors within 5–8%. The main sources of systematic error include laser and microwave intensity fluctuations, thermal gradient, and nanometer-scale inhomogeneity between the NV probe and sample interface. Nevertheless, the experimental uncertainty remains well below the magnitude of the observed magnetic signals, supporting the validity and reliability of the results.

## 4. Discussion

This work systematically applies noninvasive NV center-based quantum sensing to probe local static and dynamic magnetic properties of Tm_3_Fe_5_O_12_ thin films around their Curie temperature. The marked reduction in NV spin relaxation time (*T*_1_) observed in the critical region directly reflects enhanced spin fluctuations, consistent with established theories of diverging correlation lengths near second-order phase transitions [25,26]. By fitting the temperature-dependent relaxation rate with a Gaussian profile, we quantitatively demonstrate NV centers’ capability to detect locally amplified magnetic noise inherent to critical dynamics—offering microscopic insight into phase transition kinetics.

Electrical transport measurements reveal a pronounced resistivity upturn near 340 K, attributable to increased spin-disorder scattering, which is corroborated by magnetization and NV sensing results. This interplay highlights the intrinsic coupling between electron conduction and localized spin fluctuations [27,28]. Such findings extend our understanding of magneto-transport phenomena modulated by critical spin dynamics in high Curie temperature garnet films.

Compared to conventional bulk magnetometry (e.g., MPMS) or Hall effect measurements that provide volume-averaged or limited spatial information, NV magnetometry affords submicron spatial resolution (~360 nm) and dynamic sensitivity (~1.6 μT/√Hz), enabling direct visualization of local critical phenomena inaccessible to conventional techniques. We also note that magnetic force microscopy (MFM) is a widely used technique capable of nanoscale magnetic imaging with spatial resolution down to tens of nanometers [29,30]. However, MFM typically lacks quantitative field sensitivity and can be invasive to delicate magnetic samples, whereas NV magnetometry offers noninvasive, quantitative, and dynamic measurements under ambient conditions.

However, we acknowledge that our current configuration does not permit wide-field imaging or systematic spatial mapping of local magnetic fields, either laterally across the film or as a function of probe depth. Such advanced capabilities—achievable in wide-field or scanning-probe NV platforms—provide even clearer demonstrations of spatial resolution and magnetic field gradients [31,32]. Despite these limitations, our confocal NV approach allows for high-sensitivity, site-specific measurements with spatial detail several orders of magnitude finer than traditional volume-averaged methods. Future integration of advanced NV-based imaging modalities may enable real-time 2D/3D mapping of local magnetic textures and further clarify nanoscale magnetic phase phenomena.

Below is a comparison of the multidimensional performance parameters of different magnetic characterization techniques (see Table 1). The table details the detection dimensions, spatial resolution, response times, and technical advantages of MPMS, Hall effect, and NV center methods. This comparison helps readers gain a clearer understanding of the applicable scenarios and limitations of each technique.

The integration of nanoscale quantum sensing with macroscopic characterization methods opens new avenues for probing complex magnetic heterostructures. As NV sensor technology advances, future studies can further explore spin dynamics, topological magnetism, and quantum materials under diverse conditions.

## 5. Conclusions

In conclusion, this work demonstrates that diamond nitrogen-vacancy (NV) center quantum sensing offers a highly precise and sensitive platform for investigating local dynamic magnetic fluctuations in Tm_3_Fe_5_O_12_ thin films near their Curie temperature. By combining NV-based quantum magnetometry with conventional bulk techniques such as MPMS and Hall effect measurements, we reveal microscopic magnetic phase transition mechanisms typically hidden in bulk-averaged characterizations, advancing our understanding of nanoscale ferromagnetic dynamics. This study paves the way for employing quantum sensing in exploring complex magnetic heterostructures and optimizing spintronic device architectures. Future improvements in NV sensor technology and multimodal measurement integration will further enhance sensitivity, spatial resolution, and the scope of applications within condensed matter physics.

## Figures and Tables

**Table 1:** Comparison of multidimensional performance parameters of magnetic characterization techniques.

Technique	MPMS	Hall Effect	NV Centers	MFM
Detection dimension	Entire volume	Point-to-point electrode	Surface local	Surface local
Spatial resolution	Millimeter scale	Micrometer scale	~360 nm	<10 nm
Response time	Static	Static	Dynamic	Static
Information type	Average magnetization	Out-of-plane magnetization	Magnetic noise/imaging	Surface magnetic structure
Advantages	Mature, reliable, high-sensitivity	Direct electrical signal	Non-contact, high spatial resolution, and dynamic	Ultra-high spatial resolution, surface mapping

## Data Availability

The original contributions presented in this study are included in the article; further inquiries can be directed to the corresponding author.

## Abbreviations

The following abbreviations are used in this manuscript:

SQUID	Superconducting Quantum Interference Device
MFM	Magnetic force microscopy
MPMS	Magnetic Property Measurement System
